# The absence of metamictisation in natural monazite

**DOI:** 10.1038/s41598-020-71451-7

**Published:** 2020-09-07

**Authors:** Lutz Nasdala, Shavkat Akhmadaliev, Boris E. Burakov, Chutimun Chanmuang N, Radek Škoda

**Affiliations:** 1 Institut für Mineralogie und Kristallographie, Universität Wien, Althanstr. 14, 1090 Wien, Austria; 2 Institut für Ionenstrahlphysik und Materialforschung, Helmholtz-Zentrum Dresden-Rossendorf e.V., 01328 Dresden, Germany; 3 Laboratory of Applied Mineralogy and Radiogeochemistry, V. G. Khlopin Radium Institute, 28, 2nd Murinskiy Ave., St. Petersburg, 194021, Russia; 4 Department of Geological Sciences, Masaryk University, Kotlářská 2, 61137 Brno, Czech Republic

**Keywords:** Mineralogy, Solid Earth sciences

## Abstract

The actinide-containing mineral monazite–(Ce) is a common accessory rock component that bears petrogenetic information, is widely used in geochronology and thermochronology, and is considered as potential host material for immobilisation of radioactive waste. Natural samples of this mineral show merely moderate degrees of radiation damage, despite having sustained high self-irradiation induced by the decay of Th and U (for the sample studied herein 8.9 ± 0.3 × 10^19^ α/g). This is assigned to low damage-annealing temperature of monazite–(Ce) and “alpha-particle-assisted reconstitution”. Here we show that the response of monazite–(Ce) to alpha radiation changes dramatically, depending on the damage state. Only in radiation-damaged monazite–(Ce), ^4^He ions cause gradual structural restoration. In contrast, its high-temperature annealed (i.e. well crystalline) analogue and synthetic CePO_4_ experience He-irradiation damage. Alpha-assisted annealing contributes to preventing irradiation-induced amorphisation (“metamictisation”) of monazite–(Ce); however, this process is only significant above a certain damage level.

## Introduction

Monazite–(Ce), ideally CePO_4_, is the prevalent monazite-group mineral in the lithosphere. It commonly occurs as an accessory component in magmatic and metamorphic rocks and as a detrital phase in clastic sediments; particularly large crystals are found in pegmatite dikes. The monoclinic structure (space group *P*2_1_/*n*; Z = 4) of monazite–(Ce) exhibits chains of alternating, edge-sharing CeO_9_ polyhedrons and distorted PO_4_ tetrahedrons along the crystallographic *c* axis, which are cross-linked by zigzag chains of edge-sharing CeO_9_ polyhedrons along the crystallographic *a* axis^1^. Monazite–(Ce) is characterised by a wide range of chemical compositions^2–4^. A significant fraction of the Ce^3+^ is typically replaced by other LREE^3+^ (light rare earth elements; predominantly La^3+^ and Nd^3+^) and the actinides Th^4+^ and U^4+^ (even though minor amounts of U^5+^ and U^6+^ may also be present^5^). For the incorporation of actinides, charge balance is effectuated predominantly by the incorporation of Ca [2Ce^3+^ ↔ (Th,U)^4+^ + Ca^2+^; referred to as “cheralite substitution”], and/or the incorporation of Si [Ce^3+^ + P^5^  ↔ (Th,U)^4+^ + Si^4+^; referred to as “huttonite substitution”]^4,6^. Monazite–(Ce) has the ability to accommodate significant amounts of actinides, with ThO_2_ contents exceeding 20 wt%^2,7–9^, and in rare cases UO_2_ well above 10 wt%^10^. The radioactive decay of the actinides over geologic periods of time may form high amounts of radiogenic Pb. In contrast, Pb is largely rejected upon primary crystallisation of monazite–(Ce)^11^. Monazite–(Ce) contains high levels of “common” Pb only in exceptional cases^12^, in particular after being re-crystallised under high-pressure conditions^13^. The Pb has a place in the monazite structure^14^, which explains the low tendency of this element to escape from monazite–(Ce). This, in turn, substantiates the wide application of monazite–(Ce) in U–Th–Pb geochronology^4,15^. Also, the apparent radiation resistance of monazite–(Ce) and related phosphate phases has stimulated their consideration as potential, inert waste form for the immobilisation of hazardous radionuclides originating from dismantled nuclear weapons, spent nuclear fuel and other sources^16–19^.

**Figure 1:**
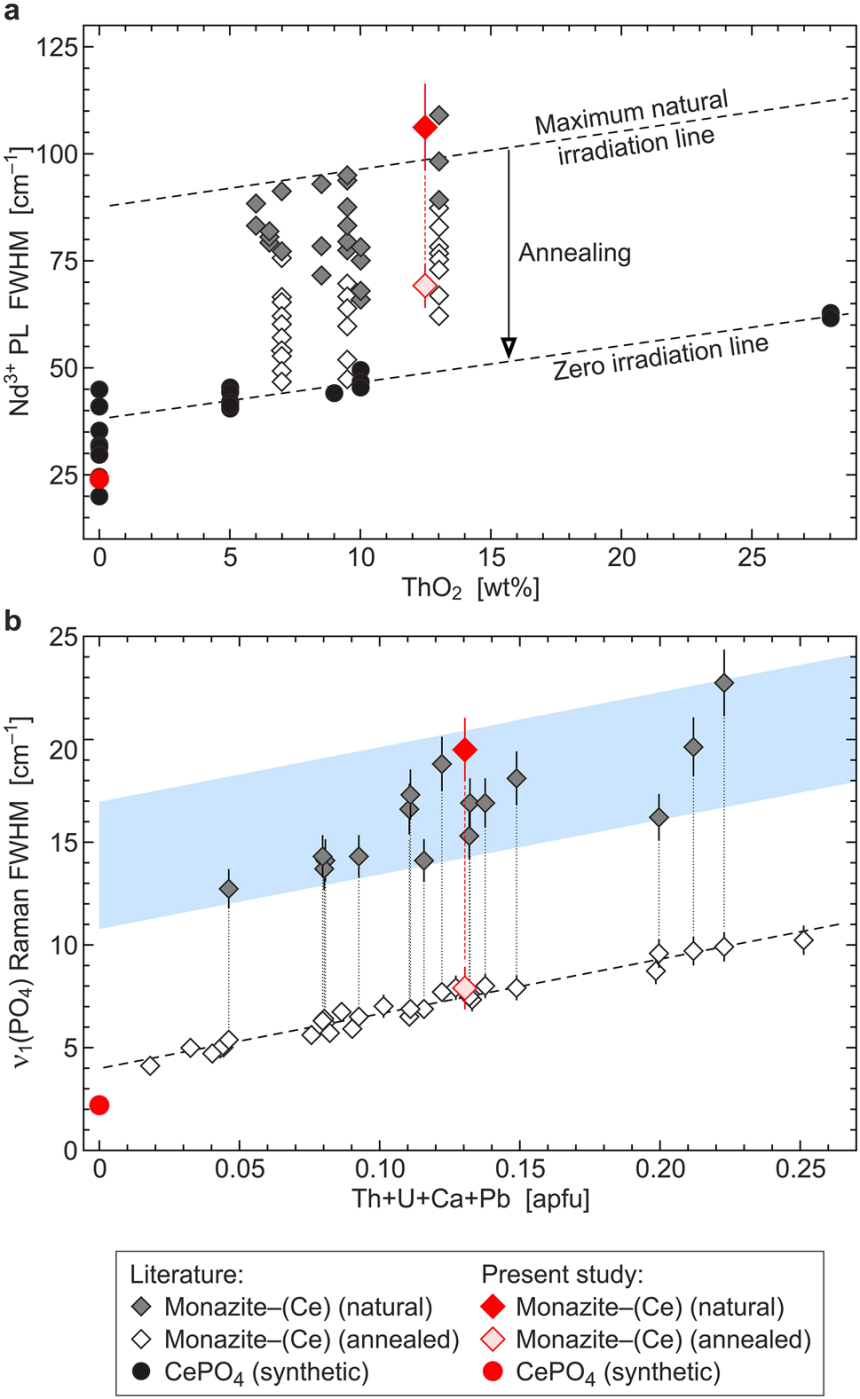
Spectroscopy-based estimation of radiation damage in monazite–(Ce). (**a**) Plot of the broadening (*FWHM* full width at half maximum) of the ~ 11,590 cm^–1^ emission line (belonging to the crystal-field-split ^4^F_3/2_ → ^4^I_9/2_ electronic transition of Nd^3+^) against the ThO_2_ content (redrawn from Ref.^29^). Fully annealed natural monazite–(Ce) and synthetic Th-doped CePO_4_ define a “zero irradiation line”. Note the experiential “maximum natural irradiation line” of Ref.^29^. (**b**) Broadening of the main PO_4_ stretching Raman band depending on radiation damage and non-formula cations (redrawn from Ref.^20^). Data of annealed samples analogously define a “zero irradiation line”. All samples, originating from locations worldwide, were found to represent similarly moderate degrees of radiation damage. We have underlain the plot with a blue bar to visualise that no natural sample falls short of a minimum, and none exceeds a certain maximum, level of disorder.

Despite suffering high self-irradiation doses over geologic periods of time (typically on the order of 10^19^–10^20^ α/g^20^), monazite–(Ce) in nature does not become metamict. This term goes back to “metamikte”, which was introduced by Waldemar Christofer Brøgger^21^ to describe a special class of amorphous materials that nevertheless show well-shaped crystal forms. As early as in 1914, it was suggested that the metamictisation process is caused by corpuscular radiation^22^. Nowadays the term metamict is used independently from the outer crystal shape; it denominates initially crystalline minerals that were transformed, due to the impact of radioactivity, to a glass-like, aperiodic state^23,24^. Even though it has been stated occasionally^25,26^ that monazite–(Ce) “rarely” does become metamict, we were unable to find any confirmed reference for a completely aperiodic, natural specimen of this mineral. Instead, natural monazite–(Ce) always seems to be crystalline^8,27,28^, with the vast majority of samples being characterised by comparably similar, moderate degrees of radiation damage^20,29^ (Fig. 1). This appears to be in striking contrast to the fact that monazite–(Ce) is prone to radiation damage and can be amorphised by ion irradiation in the laboratory^28,30–34^.

The reasons for why extensive corpuscular self-irradiation in nature does not transfer monazite–(Ce) to a completely aperiodic state, are still controversial. It is generally agreed that radiation damage in monazite–(Ce) self-anneals over geological periods of time at fairly low temperatures^8,25,35^, thus preventing the accumulation of significant amounts of damage. In addition, it has been proposed^33^ that annealing of radiation damage in monazite–(Ce) may be induced by alpha particles. More recently it was shown that pre-existing radiation damage in REEPO_4_ may indeed recover upon irradiation with ^4^He ions of MeV energy^36^. The relevance of the proposed alpha-annealing process is supported by the observation that ^238^Pu-doped, monazite-structured LaPO_4_ was found to remain crystalline even after sustaining high self-irradiation^33,37–39^. In contrast, annealing effects of alpha particles are questioned by self-irradiation-induced amorphisation of monazite-structured ^238^PuPO_4_^37^ and ^241^AmPO_4_^40^, and the observation that synthetic CePO_4_ is prone to He-irradiation damage^41^.

Here we constrain the effect of alpha particles on radiation damage in monazite–(Ce) by irradiation experiments with 7.7 MeV ^4^He ions. Such light ions transfer the vast majority of their energy to the target via electronic interactions (lattice ionisation). Nuclear interactions—that is, atomic knock-ons creating Frenkel-type defect pairs—occur predominantly after the ions are slowed down significantly through ionisation losses, near the far end of the ion trajectory^41,42^. This narrow depth range is commonly referred to as the Bragg peak of damage. The significant difference in depth distributions of electronic and nuclear interactions of ^4^He ions and target atoms may allow one to unravel causes of certain irradiation effects^43^. In the present study we use micro-spectroscopy as a high-resolution tool for estimating radiation damage in monazite–(Ce) and apply hyper-spectral line-scanning to obtain depth profiles of irradiation-induced changes.

## Methods

### Samples and preparation

We have investigated a monazite–(Ce) crystal (~ 20 mm size) of incarnadine to orange brown colour, originating from a pegmatite in the Iveland district, southern Norway. This natural sample was chosen as suitable candidate for ion-irradiation experiments because (i) of its size, transparency and homogeneous appearance under the optical microscope, (ii) of its apparently uniform, moderate broadening of Raman bands, and (iii) preliminary U–Pb analyses did not indicate any isotopic disturbance. Undoped CePO_4_ crystals (0.5–1 mm size) were grown from a Li–Mo flux consisting of 84 mol% MoO_3_ and 10 mol% Li_2_MoO_4_, to which 3 mol% NH_4_H_2_PO_4_ and 3 mol% CeO_2_ was added as the P and Ce sources (details are quoted elsewhere^20^). The natural sample was cut in half with a diamond-coated high-grade steel wire (0.17 mm thickness). One half was put in a Pt crucible and annealed in air, at 1,200 °C for 96 h, for structural reconstitution (details are quoted elsewhere^34^). Analogous annealing of the synthetic CePO_4_ was considered unnecessary, as (i) the material is non-radiation-damaged and (ii) synthesis had involved elevated temperatures (≥ 1,030 °C)^20^. The un-annealed and annealed halves of the monazite–(Ce) sample, having matching faces, were placed side by side in close proximity, embedded in epoxy, and ground and polished. Another sample mount containing synthetic CePO_4_ was prepared analogously. After He irradiation, mounts were cut along the ion-irradiation direction, embedded in epoxy, and plane-parallel, doubly polished thin sections were produced. For electron probe micro-analyser (EPMA) measurements, sections were coated with carbon.

### Chemical characterisation and age determination

Element analysis of the monazite–(Ce) sample was done by X-ray spectrometry using a Cameca SX100 EPMA operated in wavelength-dispersive mode, at 15 kV and 200 nA. The comparably high beam current was chosen to improve counting statistics on Pb and hence to get a chemical age with lower standard deviation. The electron beam was defocused to a ~ 8 μm spot, to reduce the energy density impacting the sample surface. After analysis, no surface damage in the analysis spots was visible in back-scattered electrons imaging mode. The Pb–M_α_ count rates were corrected for possible interferences with Y–L_γ_ and Th–M_ζ_, and the U–M_β_ count rates were corrected for possible interferences with Th–M_γ_. X-ray lines analysed, calibrant materials and counting times are listed in Supplementary Table S1 in the Supplementary Material. More details are reported elsewhere^44^. Results of EPMA chemical analyses are presented in Supplementary Table S2, along with the chemical composition of the synthetic CePO_4_ as determined by Ref.^20^ (Supplementary Table S3; Supplementary Material). The monazite–(Ce) sample contains 12.5 ± 0.2 wt% ThO_2_, 0.30 ± 0.01 wt% UO_2_ and 0.51 ± 0.001 wt% PbO (2σ; n = 10). A CHIME^45^ Th–total U–Pb age of 888 ± 12 Ma (2σ; n = 10) was calculated from the EPMA results (Supplementary Table S4; Supplementary Material). From the present actinide concentrations (c_U_ and c_Th_ in μg/g), a time-integrated α dose (D_α_) of 8.9 ± 0.3 × 10^19^ α/g was calculated according to^46^

1 $${\text{D}}_{\alpha } = 8 \cdot \frac{{{\text{c}}_{{\text{U}}} {\text{~}} \cdot ~{\text{N}}_{{\text{A}}} \cdot ~0.9928}}{{{\text{M}}_{{238}} ~ \cdot ~10^{6} }} \cdot \left( {{\text{e}}^{{\lambda _{{238}} {\text{t}}}} - 1} \right) + 7 \cdot \frac{{{\text{c}}_{{\text{U}}} ~ \cdot ~{\text{N}}_{{\text{A}}} ~ \cdot 0.0072}}{{{\text{M}}_{{235}} ~ \cdot ~10^{6} }} \cdot \left( {{\text{e}}^{{\lambda _{{235}} {\text{t}}}} - 1} \right) + 6 \cdot \frac{{{\text{c}}_{{{\text{Th}}}} {\text{~}} \cdot ~{\text{N}}_{{\text{A}}} }}{{{\text{M}}_{{232}} ~ \cdot ~10^{6} }} \cdot \left( {{\text{e}}^{{\lambda _{{232}} {\text{t}}}} - 1} \right)$$

with N_A_ = Avogadro’s number; M_238_, M_235_ and M_232_ = atomic weights of the three parent isotopes; λ_238_, λ_235_ and λ_232_ = half-life times of the three parent isotopes; t = integration time (i.e., age of the sample).

### He-ion irradiation

The sample mounts were irradiated with ^4^He^2+^ ions using the HVEE (High Voltage Engineering Europa B.V.) 3 MV Tandetron accelerator^47^ at Helmholtz-Zentrum Dresden-Rossendorf, Germany. The irradiation fluence was 10^16^ ions per cm^2^; additional irradiations with 10^15^ and 10^17^ ions per cm^2^ were done for synthetic CePO_4_. The He-ion energy was set to 7.7 MeV, which lies well within the energy range of common alpha particles in the ^238^U, ^235^U and ^232^Th decay chains (3.9–8.8 MeV). Samples were loaded into an implantation chamber that was evacuated to ~ 3 × 10^–7^ bar, and cooled to −196 °C with liquid N_2_ to avoid any uncontrolled sample heating during the irradiation. Samples (that had random crystallographic orientation in the mounts) were irradiated perpendicular to their polished surfaces.

### Micro-spectroscopy

Laser-induced PL (photoluminescence) and Raman spectra, and line scans along the He-irradiation directions, of the samples were obtained at room temperature using a Horiba LabRAM HR Evolution dispersive spectrometer. This single-stage system has a focal length of 800 mm and is equipped with Olympus BX-series optical microscope and Peltier-cooled, Si-based CCD (charge-coupled device) detector. PL spectra were excited with the 532 nm emission of a frequency-doubled Nd:YAG laser (12 mW power behind the objective), and Raman spectra were excited with the 632.8 nm emission of a He–Ne laser (5 mW). In both cases, a 100× air objective (numerical aperture NA = 0.9) was used, and the system was operated in confocal mode. A grating with 600 grooves/mm (PL) and 1,800 grooves/mm (Raman), respectively, was used to disperse the light to be analysed. Wavenumber calibration was done using the zero-order line, the respective Rayleigh line, and emission lines of a Kr lamp. The wavenumber accuracy was better than 0.5 cm^−1^, and the FWHM (full width at half maximum) of the instrumental profile function was ~ 2 cm^−1^ (PL) and ~ 0.8 cm^−1^ (Raman), respectively. Hyperspectral (point-by-point) line scans were placed in areas in which the material appeared well transparent and virtually free of inclusions. Line scans were done in “oversampling” mode, that is, the step size (0.2 μm) was smaller than the lateral resolution^48^. After background correction, spectral fitting was done assuming pseudo-Voigt shapes of PL and Raman signals. Measured FWHM values (Γ_m_) were corrected for instrumental band broadening, and real FWHM values (Γ) were calculated, using the empirical formula^49^

2 $$\Gamma = \Gamma_{{\mathbf{m}}} - \frac{{\left( {\Gamma_{{{\mathbf{IPF}}}} } \right)^{2} }}{{0.9 \times \Gamma_{{\mathbf{m}}} + 0.1 \times \Gamma_{{{\mathbf{IPF}}}} }}$$

with Γ_IPF_ = FWHM of the spectrometer’s instrumental profile function (IPF).

### Monte Carlo simulation

The stopping of 7.7 MeV ^4^He ions irradiated into a CePO_4_ target was calculated using the SRIM (The Stopping and Range of Ions in Matter; version 2013) code^50^. Displacement threshold energies of 56 eV for Ce, 75 eV for P and 8 eV for O atoms were used. In doing so, we have adopted the threshold displacement energies obtained for monazite-structured LaPO_4_^51^, assuming that threshold displacement energies for Ce and La in the same host structure are reasonably similar. The SRIM defaults for binding energies were accepted for all atomic species. The target density was set to 8.022 × 10^22^ atoms per cm^3^, which corresponds to a mass density of 5.22 g/cm^3^. The simulation was done for 100,000 incoming He ions, for statistical precision, and has included full damage cascades (i.e. both displacements caused by irradiated He ions and displacements caused by displaced target atoms).

## Results

### Effects of ^4^He ions on synthetic CePO_4_

**Figure 2:**
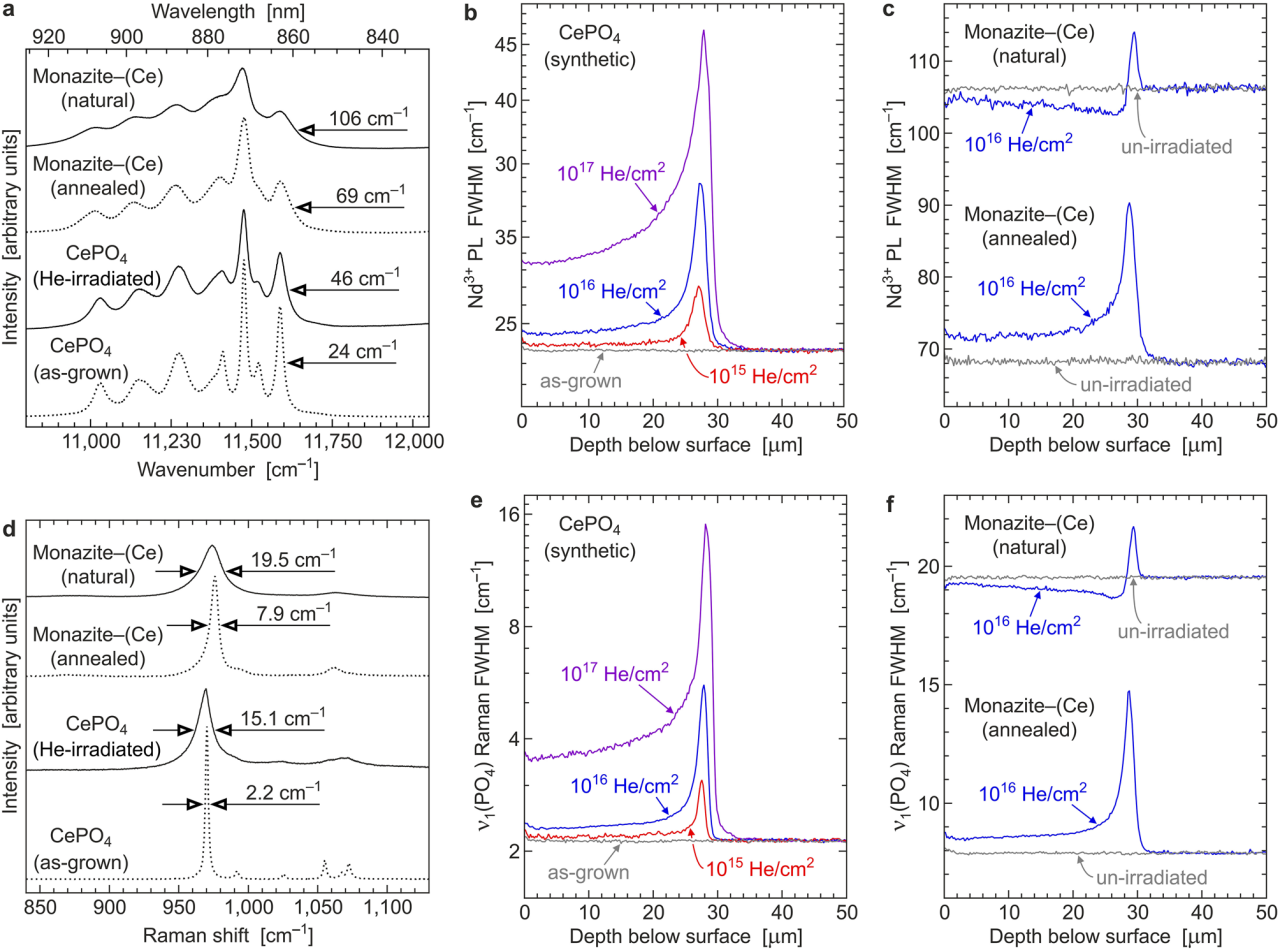
Spectra and hyperspectral line-scans. (**a**) Photoluminescence emission (532 nm excitation) of naturally self-irradiated and annealed monazite–(Ce), and un-irradiated and irradiated CePO_4_, in the near infrared range, related to the crystal-field-split ^4^F_3/2_ → ^4^I_9/2_ electronic transition of Nd^3+^^52,53^. The spectrum of ion-irradiated CePO_4_ (10^17^ He/cm^2^) was obtained within the Bragg peak (maximum line broadening at 28 μm below the surface); all other spectra represent the un-irradiated bulk (obtained at 45–50 μm below the surface). (**b**) Depth profiles of the FWHM of the ~ 11,590 cm^–1^ emission line in He-irradiated CePO_4_. (**c**) Depth profiles of the FWHM of the ~ 11,590 cm^–1^ emission line in He-irradiated monazite–(Ce). (**d**) Raman spectra (632.8 nm excitation) showing the PO_4_ stretching range^20,55^. Analysis points correspond to that of PL spectra in (**a**). (**e**) Depth profiles of the FWHM of the ~ 970 cm^–1^ Raman band in He-irradiated CePO_4_. (**f**) Depth profiles of the FWHM of the ~ 970 cm^–1^ Raman band in He-irradiated monazite–(Ce).

The impact of ^4^He ions on the structural state of synthetic (i.e., initially crystalline) CePO_4_ was first monitored using the FWHM of the ~ 11,590 cm^−1^ Stark line^29,34^ in the PL spectrum (Fig. 2a), which belongs to the crystal-field split ^4^F_3/2_ → ^4^I_9/2_ electronic transition of Nd^3+^^52,53^. This was possible because the “pure” CeO_2_ used for synthesis was minimally contaminated with other REE, resulting in CePO_4_ crystals that are unintentionally doped, among others, with trace amounts of Nd^3+^. Second, He-irradiation effects were monitored using the FWHM of the main Raman band^20,32,34,41,54^ of CePO_4_ at ~ 970 cm^−1^ Raman shift (Fig. 2d), which is assigned to the symmetric stretching of PO_4_ tetrahedrons (A_g_-type vibration^20,55^).

Hyperspectral PL and Raman line scans yielded widely similar FWHM-versus-depth patterns. For CePO_4_ irradiated with 1 × 10^15^ He/cm^2^ (Fig. 2b,e), the depth range 0–26.5 μm below the surface shows little disorder, whereas the narrow depth range 26.5–28.5 μm below the surface shows significant FWHM increases. Such depth-distribution patterns suggest the creation of damage predominantly due to nuclear interactions of He ions with lattice atoms, with the 26.5–28.5 μm depth range corresponding to the Bragg peak of atomic knock-ons^41,42^. However, observed minor FWHM increases in the 0–26.5 μm depth range cannot be explained by nuclear interactions alone and hence indicate minor contribution of electronic He–target interactions to damage creation. The verification of this contribution will require further study.

It is most remarkable that increases of the He fluence do not result in nearly equivalent, but much lesser FWHM increases (Fig. 2b,e). Even after irradiation with 1 × 10^17^ He/cm^2^, CePO_4_ has remained crystalline, with still moderate levels of radiation damage within the Bragg peak. For comparison, irradiation of quartz^56^ and diamond^57^ with only 1 × 10^16^ He/cm^2^ was found to result in the formation of a fully aperiodic (i.e., metamict) state within the Bragg peak of damage. Our observations suggest that the susceptibility of CePO_4_ to receive, and accumulate, irradiation damage depends strongly on the present damage and decreases significantly already at early stages of damage accumulation.

### Effects of ^4^He ions on monazite–(Ce)

The natural monazite–(Ce) sample investigated in the present study had accumulated moderate self-irradiation damage over geologic periods of time, which is documented by moderately broadened PL^29,34,41^ (Fig. 2a) and Raman bands^20,32,34,54^ (Fig. 2d). The significant contribution of radiation damage to the observed band broadening, in addition to “chemical band broadening” caused by short-range disorder due to elevated levels of non-formula chemical constituents^53^, is documented by significant FWHM decreases upon dry thermal annealing (grey lines in Fig. 2c,f). In the FWHM (Nd^3+^) versus ThO_2_ plot (Fig. 1a), our annealed sample plots above the “zero irradiation line”^29^ that is defined by synthetic Th-doped CePO_4_. This is explained by the broadening effect of other non-formula elements present^20,53^. In addition to moderately broadened PL lines and Raman bands of the natural sample, rather “regular” interference colours and homogeneous extinction in cross-polarised transmitted mode (not shown) indicate that (i) a moderate degree of damage is present, and (ii) the sample is still a single crystal and hence has never during its post-growth history experienced very high levels of damage. The latter is concluded because at elevated levels of radiation damage accumulation, isolated crystalline remnants in an aperiodic matrix are formed, which are likely to rotate^25,58^. This, and (unoriented) random nucleation in the amorphous volume fraction of a heavily radiation-damaged material, would have caused the formation of a polycrystalline compound, rather than a single crystal, upon thermal annealing. As it is generally the case for natural monazite–(Ce)^20^, the observed crystalline state with moderate radiation damage is in apparent contrast to the calculated α dose of 8.9 ± 0.3 × 10^19^ events per gram, which exceeds the threshold to alpha-event amorphisation by about two orders of magnitude^34^.

He-irradiation of the annealed (i.e. well crystalline) counterpart of the natural monazite–(Ce) sample has resulted in depth distribution patterns of disorder (Fig. 2c,f) that are comparable to those observed in irradiated synthetic CePO_4_ (Fig. 2b,e). This suggests that, analogous to un-doped CePO_4_, and in spite of its non-formula chemical composition, crystalline monazite–(Ce) is prone to He-irradiation damage. More detailed irradiation experiments, of samples with various compositions, will be needed to study as to which degree chemical deviations in the target may increase or decrease the extent of He-irradiation effects.

**Figure 3:**
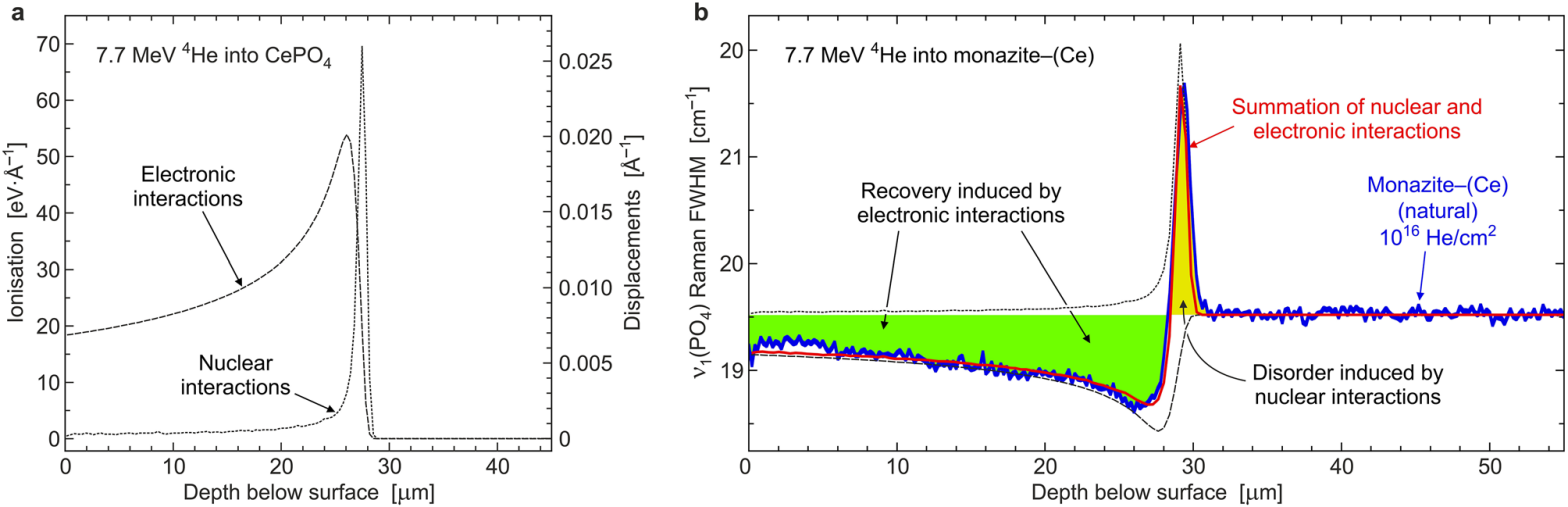
Interpretation of irradiation effects in monazite–(Ce). (**a**) Depth profiles of electronic energy losses (target ionisation) and nuclear energy losses (atomic displacements) per 7.7 MeV ^4^He ion in a CePO_4_ target, as predicted by Monte Carlo simulation (SRIM-2013 software package^50^; https://www.srim.org/). (**b**) The two distribution curves are fitted to the Raman depth profile in He-irradiated monazite–(Ce) (Fig. 2f), under assumption of 6% greater penetration depth in the natural sample, compared to CePO_4_. Electronic interactions of irradiated ^4^He ions with lattice atoms cause defect recombination (at 0–28 μm below the surface; green) whilst nuclear energy losses create additional damage (at 28–30 μm below the surface; yellow).

The natural (i.e., un-annealed) sample, in contrast, exhibited a substantially different response to He irradiation. There is a comparably weak Bragg peak of damage. Its location at 28–30 μm below the surface (i.e., about 1.5 μm deeper below the surface, compared to CePO_4_) is explained by somewhat deeper He penetration, caused by the target’s deviating chemical composition and slight volume expansion und corresponding mass density decrease at moderate radiation damage. In the depth range 0–28 μm below the surface, however, there are significant FWHM decreases (Fig. 2c,f), indicating reduction of the disorder and structural reconstitution. The latter is assigned to recovery induced by electronic interactions of He ions with lattice atoms. In conclusion, whereas damage creation as caused by nuclear interactions prevails only within the Bragg peak, most of the irradiated volume has experienced gradual recovery as caused by electronic interactions. We support this interpretation by presenting a fit of the summation of nuclear and electronic interactions as predicted by Monte Carlo simulation (Fig. 3a) to the Raman depth profile (Fig. 3b).

## Implications

Corpuscular irradiation (including self-irradiation) of minerals and materials may have decidedly diverse effects. Results depend first on the target irradiated, including its crystal structure^59,60^, chemical composition^61^ and crystal size^62,63^. Second, irradiation effects in a given target depend on the type of radiation, in particular ion mass and energy, and the ratio of nuclear and electronic energy losses in the target^33,64^. Possible results of the electronic stopping of ions (which comprises the vast majority of the ion energy) in the target irradiated range from the creation of damage^64–66^, to damage annealing^67–69^ Our results demonstrate that the structural state is in crucial control of He-irradiation effects in monazite–(Ce), as even moderate differences in the present degree of radiation damage may cause vast differences in the response to further corpuscular irradiation. Up to mild levels of existing radiation damage, electronic interactions of 7.7 MeV ^4^He ions seem to contribute secondarily to the creation of additional damage. In contrast, already at moderate levels of pre-existing radiation damage, electronic interactions of 7.7 MeV ^4^He ions cause significant damage annealing whose extent exceeds damage that is generated simultaneously within the Bragg peak by nuclear energy losses.

Our results explain the apparently contrasting observations of damage creation in crystalline^41^, and structural recovery in amorphised^36^, REEPO_4_ upon irradiation with ^4^He ions. Alpha-assisted annealing of radiation damage in monazite–(Ce) has been proposed before^33,36^; however, these proposals were guilty of too much simplification and were not able to explain why alpha particles may in some cases heal and in other cases create radiation damage in monazite–(Ce). We show that the alpha-assisted annealing process is not a general feature of monazite-structured REEPO_4_ but only becomes relevant above a certain level of accumulated radiation damage. We conclude that in natural monazite–(Ce), initial self-irradiation during comparably short times creates moderate levels of damage (mainly by recoils of heavy daughter nuclei upon emission of an alpha particle^41,58^), which then progressively prevents further damage accumulation and favours annealing. Depending on the respective milieu conditions (especially the temperature), an equilibrium between damage creation caused by self-irradiation on the one hand, and both thermal and alpha-particle-induced annealing on the other hand, will be achieved over geologic periods of time, resulting in a henceforward more or less constant degree of radiation damage. This interpretation explains the general observations that, except from very young samples, natural monazite–(Ce) does not fall short of a certain minimum level of radiation damage^20^, and natural monazite–(Ce) does not exceed a certain maximum level of radiation damage^20,29^ (Fig. 1).

The general behaviour of monazite–(Ce) to adjust itself, under geological milieu conditions, to a moderately radiation-damaged state has implications for petrogenesis, geochronology and thermochronology, whose detailed discussion is beyond the scope of the present study. To quote two examples, moderate radiation damage—that virtually seems always present in natural monazite–(Ce)—causes higher dissolution rates of this mineral, compared to its (chemically equivalent) annealed and structurally reconstituted counterparts^26^. Also, moderate radiation damage may impede He diffusivity in monazite–(Ce)^70^.

On the contrary, we do not hold to the opinion that the continuous self-annealing of monazite-structured REEPO_4_ is a quality factor that promotes these materials as nuclear waste forms, by keeping them in a crystalline and hence “supposedly robust” state. It is well known that monazite–(Ce) in nature may undergo secondary chemical degradation^71–75^. Not only the actinides Th and U but also REE can be mobilised from this mineral and fractionated, even at low temperatures^44,76^. Similarly, phosphatic wasteforms are known to undergo degradation that involves REE^3+^ and U^4+^ and Th^4+^ mobilisation^77^. In spite of retaining high crystallinity, EuPO_4_ doped with 4.9 wt% of ^238^Pu (6 wt% of all Pu isotopes) was found to undergo intense fracturing and form a surficial precipitate shell of “rhabdophane” (EuPO_4_ ∙ nH_2_O) during 18 years of self-irradiation^78,79^. Wasteform materials may interact with the stainless steel of canister walls, for instance under conditions of hot isostatic pressing. Such interaction zones tend to be much more extensive in the case of phosphate-based^80^ compared to oxide-based^81^ wasteforms. Also, in case of phosphate compounds that are solid solutions [such as (La,Pu)PO_4_], annealing following self-irradiation may not result in the restoration of the initial compound but in phase separation^79^. The above observations may raise doubts on the general suitability of monazite-structured REEPO_4_ in immobilising radionuclides in a waste repository. They imply that numerous repetitions of damage creation and damage annealing experienced by self-irradiating orthophosphate do indeed result in maintaining crystallinity but, nevertheless, induce instability and enhance alteration. Focussing on potential wasteform materials that retain chemical durability in spite of becoming metamict^81–83^ may appear more worthwhile.

We therefore see the main implications in the Earth sciences. The sensitivity of properties of monazite–(Ce) to rather minor variations of this mineral’s structural state limits the relevance and applicability of studies conducted using synthetic samples, or thermally annealed or ion-beam amorphised analogues of natural samples. It needs to be considered cautiously in the interpretation of results that annealing of monazite–(Ce) at elevated temperatures during long-term diffusion^84,85^ and other experiments may provoke results that apply to recrystallised, but not necessarily to natural (i.e., naturally radiation-damaged) monazite–(Ce). In irradiation experiments, uncontrolled sample heating needs to be avoided. Also, possible direct effects of irradiations (such as ^3^He implantation^86^ or high-energy proton irradiation to induce ^3^He formation^87^, done to study He diffusivity) on the structural state of monazite–(Ce) need to be considered circumspectly. It seems expedient to always analyse the sample’s structural state before and after conducting experiments. We will gain further insight into the long-term behaviour of monazite–(Ce) in the lithosphere, and avoid possible bias in the interpretation of petrogenesis and geo- and thermochronology results, only if experiments are conducted using moderately radiation-damaged samples whose structural state corresponds to that of natural monazite–(Ce).

## Supplementary information

Supplementary Information.

## Data Availability

All data used in this study are available in the Supplementary Material. In addition, the spectroscopic raw data are available from the corresponding author upon reasonable request.
