# Supplementary material for: The absence of metamictisation in natural monazite
Source: Sci Rep. 2020 Sep 7;10:14676. doi: 10.1038/s41598-020-71451-7 (PMC7477544; doi:10.1038/s41598-020-71451-7)
Supplement: Supplementary file 1 — Supplementary Information. [file 41598_2020_71451_MOESM1_ESM.pdf]

Supplementary material accompanying the article

“The absence of metamictisation in natural monazite”

by Lutz Nasdala, Shavkat Akhmadaliev, Boris E. Burakov, Chutimun Chanmuang N.✉ & Radek Škoda
✉ E-mail: chutimun.chanmuang@univie.ac.at

Table S1: EPMA analytical details

Element*	Line analysed	Calibrant material	Counting time (s)		Detector crystal
			Peak	Background	
Si	K_α	natural wollastonite	40	20	TAP
P	K_α	natural fluorapatite	10	5	PET
Ca	K_α	natural fluorapatite	10	5	LPET
Y	L_α	synthetic Y-Al garnet	40	20	TAP
La	L_α	synthetic $LaPO_4$	20	10	PET
Ce	L_α	synthetic $CePO_4$	20	10	PET
Pr	L_β	synthetic $PrPO_4$	20	10	LLIF
Nd	L_β	synthetic $NdPO_4$	20	10	LLIF
Sm	L_β	synthetic $SmPO_4$	30	15	LLIF
Gd	L_β	synthetic $GdPO_4$	30	15	LLIF
Tb	L_α	synthetic $TbPO_4$	30	15	LLIF
Dy	L_α	synthetic $DyPO_4$	30	15	LLIF
Er	L_α	synthetic $ErPO_4$	30	15	LLIF
Pb	M_α	natural vanadinite	200	100	LPET
Th	M_α	synthetic ThO_2	60	30	LPET
U	M_β	U metal	60	30	LPET

* S, Fe, As, Sr, Y and Eu were also sought but not detected.

Table S2: Results of chemical analyses of the natural monazite–(Ce) sample (present study)

Analysis #	SiO_2	P_2O_5	CaO	Y_2O_3	La_2O_3	Ce_2O_3	Pr_2O_3	Nd_2O_3	Sm_2O_3	Gd_2O_3	Tb_2O_3	Dy_2O_3	Er_2O_3	PbO	ThO_2	UO_2	Total
Major oxides (EPMA results; wt%):																	
1	3.11	25.27	0.136	2.97	6.73	22.21	3.35	14.12	4.31	2.25	0.235	0.693	0.095	0.503	12.33	0.305	98.67
2	3.04	25.94	0.136	3.02	6.80	22.25	3.45	14.36	4.34	2.19	0.239	0.685	0.110	0.510	12.17	0.313	99.61
3	3.14	25.97	0.135	2.98	6.74	22.17	3.45	14.34	4.41	2.27	0.253	0.674	0.121	0.518	12.42	0.308	99.98
4	3.09	25.71	0.136	3.03	6.76	22.19	3.46	14.36	4.39	2.25	0.226	0.670	0.106	0.498	12.33	0.301	99.61
5	3.17	25.79	0.141	2.97	6.75	22.07	3.37	14.39	4.32	2.21	0.236	0.663	0.143	0.522	12.52	0.291	99.74
6	3.09	25.83	0.139	3.00	6.79	22.17	3.44	14.46	4.45	2.20	0.241	0.662	0.112	0.516	12.33	0.280	99.81
7	3.25	25.77	0.136	2.94	6.66	21.98	3.45	14.46	4.35	2.20	0.240	0.687	0.112	0.521	12.79	0.293	99.90
8	3.14	25.88	0.137	3.00	6.77	22.13	3.44	14.34	4.39	2.27	0.230	0.654	0.102	0.509	12.52	0.283	99.91
9	3.27	25.66	0.137	2.97	6.76	21.96	3.45	14.37	4.38	2.18	0.270	0.642	0.124	0.524	12.84	0.286	99.89
10	3.21	25.43	0.143	2.98	6.77	22.08	3.48	14.35	4.38	2.18	0.262	0.682	0.117	0.519	12.62	0.305	99.63
Mean	3.15	25.73	0.138	2.99	6.75	22.12	3.43	14.36	4.37	2.22	0.243	0.671	0.114	0.514	12.48	0.297	99.67
2σ	0.07	0.22	0.003	0.03	0.04	0.10	0.04	0.09	0.04	0.04	0.014	0.016	0.013	0.009	0.22	0.011	0.38
Mean chemical formula (apfu):*																	
	Si	P	Ca	Y	La	Ce	Pr	Nd	Sm	Gd	Tb	Dy	Er	Pb	Th	U	Ca+Pb+Th+U
	0.127	0.881	0.006	0.064	0.101	0.328	0.051	0.207	0.061	0.030	0.003	0.009	0.001	0.006	0.115	0.004	0.131

* Calculated based on four oxygen atoms per formula unit (apfu).

Table S3: Results of chemical analyses of the synthetic $CePO_4$ sample (means of n = 8; from Ruschel et al. 2012)

SiO_2	P_2O_5	CaO	Y_2O_3	La_2O_3	Ce_2O_3	Pr_2O_3	Nd_2O_3	Sm_2O_3	Eu_2O_3	Gd_2O_3	ThO_2	UO_2	Total
Major oxides (EPMA results; wt%):													
bdl	29.7	bdl	bdl	0.20	69.7	bdl	bdl	0.11	bdl	bdl	bdl	bdl	99.71
Mean chemical formula (apfu):*													
Si	P	Ca	Y	La	Ce	Pr	Nd	Sm	Eu	Gd	Th	U	
0.000	0.993	0.000	0.000	0.003	1.008	0.000	0.000	0.001	0.000	0.000	0.000	0.000	

* Calculated based on four oxygen atoms per formula unit (apfu).

bdl = below the EPMA detection limit

Reference:

Ruschel, K., Nasdala, L., Kronz, A., Hanchar, J. M., Többens, D. M., Škoda, R., Finger, F. & Möller, A. A Raman spectroscopic study on the structural disorder of monazite–(Ce). *Mineral. Petrol.* **105**, 41–55 (2012).

Table S4: Determination of CHIME Th–total U–Pb age of the natural monazite–(Ce) sample (present study)

Analysis #	Pb (wt%)[a]	Pb 3σ error (wt%)	Th (wt%)	U (wt%)[a]	Th* (wt%)[b]	Age (Ma)	Age 2σ error (Ma)
1	0.467	0.017	10.834	0.406	11.687	877	32
2	0.474	0.017	10.691	0.411	11.566	899	32
3	0.481	0.018	10.913	0.410	11.776	897	34
4	0.462	0.017	10.831	0.403	11.675	870	32
5	0.485	0.018	11.002	0.396	11.817	901	33
6	0.479	0.017	10.832	0.384	11.616	905	32
7	0.484	0.018	11.236	0.401	12.057	882	33
8	0.472	0.017	10.999	0.389	11.792	880	32
9	0.487	0.018	11.286	0.395	12.086	885	33
10	0.482	0.017	11.092	0.409	11.945	886	31
Mean	0.477		11.092	0.400	11.802	888	
2σ	0.008		0.190	0.009	0.179	12	

[a] The Pb–M_α count rates were corrected for possible interferences with Y–L_γ and Th–M_ζ., and the U–M_β count rates were corrected for possible interferences with Th–M_γ.

[b] Th* (Th plus the equivalent of U) was calculated according to

$ThO_2^* = ThO_2 + UO_2\ (W_{Th}/W_U) \times [\{\exp\lambda_{235}t) + 137.88\exp(\lambda_{238}t)\}/138.88-1]\ /\ \{\exp(\lambda_{232}t)-1\}$(Suzuki and Kato 2008),

where W = molecular weights in g (W_{Th} = 264; W_U = 270).

Reference:

Suzuki, K. & Kato, T. CHIME dating of monazite, xenotime, zircon and polycrase: Protocol, pitfalls and chemical criterion of possibly discordant age data. *Gondwana Res.* **14**, 569–586 (2008).
